# Comparison of fractionation proteomics for local SWATH library building

**DOI:** 10.1002/pmic.201700052

**Published:** 2017-08-22

**Authors:** Elisabeth Govaert, Katleen Van Steendam, Sander Willems, Liesbeth Vossaert, Maarten Dhaenens, Dieter Deforce

**Affiliations:** ^1^ Laboratory of Pharmaceutical Biotechnology Ghent University Ghent Belgium

**Keywords:** Comparative study, Fractionation proteomics, hESC, Library building, SWATH‐MS

## Abstract

For data‐independent acquisition by means of sequential window acquisition of all theoretical fragment ion spectra (SWATH), a reference library of data‐dependent acquisition (DDA) runs is typically used to correlate the quantitative data from the fragment ion spectra with peptide identifications. The quality and coverage of such a reference library is therefore essential when processing SWATH data. In general, library sizes can be increased by reducing the impact of DDA precursor selection with replicate runs or fractionation. However, these strategies can affect the match between the library and SWATH measurement, and thus larger library sizes do not necessarily correspond to improved SWATH quantification. Here, three fractionation strategies to increase local library size were compared to standard library building using replicate DDA injection: protein SDS‐PAGE fractionation, peptide high‐pH RP‐HPLC fractionation and MS‐acquisition gas phase fractionation. The impact of these libraries on SWATH performance was evaluated in terms of the number of extracted peptides and proteins, the match quality of the peptides and the extraction reproducibility of the transitions. These analyses were conducted using the hydrophilic proteome of differentiating human embryonic stem cells. Our results show that SWATH quantitative results and interpretations are affected by choice of fractionation technique. Data are available via ProteomeXchange with identifier PXD006190.

**Abbreviations**
DDA: data‐dependent acquisition
DIA: data‐independent acquisition
Diff: differentiated
GP: gas phase
hESC: human embryonic stem cells
RA: retinoic acid
SWATH: sequential window acquisition of all theoretical fragment ion spectra
TEABC: triethylammonium bicarbonate buffer
Undiff: undifferentiated

## 1 Introduction

To date, data‐dependent acquisition (DDA) and MRM are the most popular acquisition types in MS‐based proteomic analyses 2, 3. The first, untargeted approach because of high throughput and broad proteome coverage and the latter, targeted approach because of excellent quantification accuracy and large dynamic range. However, in DDA the selection of peptide precursors for fragmentation is semi‐stochastic, intensity‐based, and limited to a predefined number of precursors 2, 4, 5. Consequently, reproducible identification and especially MS/MS‐based quantification of peptides and proteins across multiple samples is hampered. Data‐independent acquisition (DIA) is gaining increasing interest in the MS‐based proteomics community. DIA does not rely on the information obtained from the MS survey scan for precursor selection and fragmentation, but fragments all peptides within a sample 6.

**Significance of the study**

A reference library of DDA runs is typically used for identification of the quantitative data obtained in DIA mode by means of SWATH‐MS. These reference libraries are often created locally because of good retention time alignment and ion pattern matching. How local or repository‐based reference libraries affect SWATH performance for proteomic studies has recently been published 1. However, an evaluation of local libraries with different size obtained by different fractionation methods is currently lacking. Here we compare the SWATH performance of local reference libraries created with protein fractionation by SDS‐PAGE, peptide fractionation by high‐pH RP‐HPLC and MS‐acquisition fractionation by GP fractionation against a standard library of replicate DDA runs of the same sample. These comparisons highlight the benefits and drawbacks of each method that are needed to determine which SWATH workflow is optimal.

Sequential window acquisition of all theoretical fragment ion spectra (SWATH) 7 uses DIA data for quantification, and DDA data for identification. All the peptides are continuously fragmented with stepped *m/z* windows 7, 8 but a beforehand established reference library is typically used to identify the fragment ions from the mixed MS/MS spectra for the subsequent in silico MRM‐like quantitative data analysis. Despite the fact that bioinformatics approaches that eliminate the use of reference libraries for the interpretation of mixed MS/MS spectra from DIA experiments are being developed 1, 9, 10, the generation of comprehensive libraries is currently still a primary task for SWATH users 1, 3, 11. These reference libraries can be (i) created locally using replicate DDA runs or by fractionation, (ii) retrieved from community data repositories such as SWATHatlas (http://www.swathatlas.org/) or (iii) generated by a combination of both in an extended library 1, 12, 13. A limitation of the use of public data repositories is the variation in fragmentation patterns and retention time information according the different experimental conditions used in terms of chromatography and MS 1, 3, 14. For that reason, local libraries display the best retention time alignment and ion pattern matching, thereby guaranteeing high peptide identification success because of sample and instrument specificity 1, 3. However, their creation is sample‐ and time‐consuming.

Thus, whether used as a standalone library or combined with a public library in an extended library, SWATH users typically aim at a high degree of proteome coverage in their local library 1, 3, 11. The library size can be increased by several means. First, different peptides can be selected for fragmentation by repeated DDA injection because of the semi‐stochastic nature of DDA 2. Second, competition for precursor selection can be reduced. This includes improved 1D strategies such as decreasing gradient slope and increasing run time 4, as well as 2D strategies comprising fractionation of the sample prior to LC‐MS/MS analysis 2, 4. While low‐pH RP‐HPLC is widely used as the final fractionation step prior to MS‐acquisition, a myriad of techniques can be applied in the first dimension of separation. At the protein level, low‐pH RP‐HPLC, SEC‐LC, ion exchange chromatography, IEF, SDS‐PAGE and 2DE are the most commonly used methods 15. At the peptide level, ion exchange chromatography, IEF, high‐pH RP‐HPLC and hydrophilic interaction liquid chromatography are popular 16. At the MS‐acquisition level, gas phase (GP) fractionation 5 can be performed in which the mass spectrometer focuses on a part of the *m/z* range in each DDA run, fractionating peptide ions based on their *m/z* value.

In this study, we investigate how local libraries created using different fractionation proteomics affect SWATH performance. As a model system, Oct4‐eGFP knock‐in human embryonic stem cells (hESC) were used from which the hydrophilic proteome was extracted 17. The choice of this protocol derives from the fact that it introduces little variation as it consists of only a single step which is favorable for label‐free quantification 18, 19. Therefore, such a protocol is beneficial if one primarily cares about relative differences of quantitative DIA data and absolute numbers are of less importance, as is the case for this SWATH study. The standard library is defined as replicate DDA injection (Lib_DDA), and is compared to protein fractionation by means of SDS‐PAGE (Lib_Gel), peptide fractionation by means of high‐pH RP‐HPLC (Lib_RPRP) and MS‐acquisition fractionation by means of GP fractionation (Lib_GP). Because an increased library is only valuable if the peptides can be qualitatively (< 1% FDR) extracted from the DIA data and can be robustly quantified, the influence of the different libraries on SWATH performance was evaluated. We assessed (i) the number of peptides and proteins extracted with each reference library, (ii) the match quality of the targeted peptides expressed as the SWATH score, (iii) the extraction reproducibility of the transitions, and (iv) the consistency to detect differentially abundant proteins. We show that the increase in the number of extracted peptides and proteins from the SWATH data is not always proportional to the increase in library size attained by fractionation. Moreover, the transitions that support the peptides and proteins are low abundant and have higher CVs.

## 2 Materials and methods

### 2.1 Cell culture, sample collection, and protein extraction

The hESC WA01 Oct4‐eGFP knock‐in reporter cell line (WiCell) was cultured feeder‐free on Vitronectin XF coating (Primorigen Biosciences) in Essential 8 Medium (Life Technologies) under 5% O_2_ and 5% CO_2_ at 37°C. Every two to three days, cultures were split by means of EDTA‐passaging according to the manufacturer's protocol (Life Technologies). Karyotype analysis was performed prior to the differentiation experiment, as was the verification that the culture was free of Mycoplasma contamination (data not shown). On day three after splitting, the medium was changed to differentiation medium containing knock‐out DMEM supplemented with 2 mM L‐glutamine, 1% non‐essential amino acids, 20% knock‐out serum replacement, 100 U/ml penicillin, 100 μg/ml streptomycin and 2 μM retinoic acid (RA), and cells were subsequently cultured for another thirteen days. Cells were isolated from five biological replicates at the beginning of the experiment and from five biological replicates after thirteen days of differentiation using 0.25% trypsin‐EDTA. Subsequently, cells were washed with 1× PBS and split in two. Oct4‐eGFP levels were monitored using 2 × 10^5^ cells with an FC500 instrument (Beckman Coulter) and data was analyzed with FlowJo Analysis software. The rest of the cells was used to prepare cell lysates using R1‐buffer (Readyprep Sequential Extraction Kit, Bio‐Rad) containing 40 mM Tris pH 8.0, 1/100 Halt Protease and Phosphatase Inhibitor Cocktail (Thermo Fischer, cat. number 78 441) and 1/1000 Benzonase Nuclease (Sigma‐Aldrich, cat. number E1014‐25KU) at 1–10 × 10^6^ cells/mL. After thorough vortexing and five minutes of sonication, cells were incubated at room temperature for 30 minutes. After centrifugation at maximum speed, the supernatant was transferred to new Eppendorf protein Lobind tubes 17. The total protein concentration of the ‘R1‐extract’ was quantified using the Bradford Assay (Bio‐Rad) with BSA as the standard.

### 2.2 In‐solution digestion

The pools for library building using replicate DDA injection, high‐pH RP‐HPLC and GP fractionation, as well as the biological replicate samples for SWATH measurement were in‐solution digested as follows. Proteins were vacuum‐dried, resuspended in 500 mM TEABC, reduced for 1 h at 60°C with 100 mM DTT and alkylated for 10 min at room temperature with 200 mM methyl methanethiosulfonate. Next, samples were digested overnight at 37°C with trypsin/Lys‐C (Promega) at a protein:enzyme ratio of 25:1 in 500 mM TEABC supplemented with 1 mM CaCl_2_ and 5% ACN 20.

### 2.3 SDS‐PAGE fractionation and in‐gel digestion

Vacuum‐dried samples were resuspended in 2× Laemmli buffer (4% SDS, 20% glycerol and 10% beta‐mercaptoethanol in 50 mM Tris (pH 6.8)) and separated on a 10% TGX‐gel (Bio‐Rad). In‐gel digestion was performed as described before 20. The gel images and the MS1 profiles of the fractionation samples can be found in Supporting Information page 3–4.

### 2.4 High‐pH RP‐HPLC fractionation

High‐pH RP‐HPLC fractionation was performed using an XBridge Peptide BEH C18 Column (300 Å, 5 μm, 1 mM × 50 mm, Waters) on an Ultimate 3000 HPLC (Dionex) operating at 10 μL/min. Buffer A consisted of 10 mM ammonium formate pH 10 and buffer B of 100% ACN. Peptides were fractionated by a five‐step gradient (60 min/step): 15% B, 25% B, 30% B, 40% B and 80% B. Three fractions were collected for each step. Nonadjacent fractions were pooled based on a pre‐run in five final fractions in order to obtain maximum orthogonality with the subsequent low‐pH RP‐HPLC separation. MS1 profiles of the fractionation samples can be found in Supporting Information page 5.

### 2.5 GP fractionation

Progenesis QIP (Nonlinear Dynamics, Waters) offers direct support for optimization of GP fractionation. Based on the DDA replicate injection runs as pilot samples, the MS *m/z* range (300–1250 *m/z*) was divided in following five *m/z* ranges containing approximately the same number of peptides: 300–410, 410–490, 490–580, 580–700, 700–1250. More details can be found in Supporting Information Table 1 and Supporting Information page 6–7.

### 2.6 LC‐MS

Peptides were dissolved in 0.1% formic acid in HPLC‐grade water (buffer A). Fifty femtomole of Beta‐Galactosidase (Sciex), MassPREP Digestion Standard Mix 1 (Waters), Hi3 Ecoli Standard (Waters) and 0.1 μL iRT peptides (Biognosys) were spiked into each sample. iRT standard peptides are designed to normalize retention time variations. Fifteen micrograms and 7.5 μg of sample was loaded per injection for DDA and SWATH acquisition, respectively. MS analyses (DDA and SWATH) were performed on a TripleTOF 5600 MS (Sciex) fitted with a DuoSpray ion source in positive ion mode, coupled to an Eksigent NanoLC 400 HPLC system (Sciex). Peptides were separated on a microLC YMC Triart C18 column (id 300 μm, length 15 cm, particle size 3 μm) at a flow rate of 5 μL/min by means of trap‐elute injection (YMC Triart C18 guard column, id 500 μm, length 5 mm, particle size 3 μm). Elution was performed using a gradient of 4–45% buffer B (0.1% formic acid, 5% DMSO in 80% ACN) over 120 min. Ion source parameters were set to 5.5 kV for the ion spray voltage, 30 psi for the curtain gas, 13 psi for the nebulizer gas and 80°C as temperature. For DDA, a 4.30 s instrument cycle was repeated in high sensitivity mode throughout the whole gradient, consisting of a full scan MS spectrum (300–1250 *m/z*) with accumulation time of 0.25 s, followed by 20 MS/MS experiments (65–1600 *m/z*) with 0.20 s accumulation time each, on MS precursors with charge state 2 to 5+ exceeding a 250 cps threshold. Rolling collision energy was used as suggested by the manufacturer and former target ions were excluded for 15 s.

For SWATH, the TripleTOF 5600 system was set up in the same manner as described above with identical chromatographic conditions. Ninety six precursor isolation windows (Supporting Information Table 2) were defined using the SWATH Variable Window Calculator (Sciex) based on precursor *m/z* frequencies in the DDA samples, with a minimum window width of 3 *m/z*. The accumulation time was set to 0.25 s for the MS scan (300–1250 *m/z*) and 0.025 s for the MS/MS scans (100–1500 *m/z*). Collision energies applied for each window were calculated using rolling collision energy based on the *m/z* range of each SWATH and a charge 2+ ion, with a collision energy spread of five. The total cycle time was 2.70 s.

### 2.7 Database searching and peak extraction

PeakView software (Sciex) was used to create *.mgf files from the DDA data. For each library, a merge search of the ten DDA files was performed by MASCOT Daemon (Matrix Science, version 2.5.1) against a human database (reviewed protein database downloaded from Swissprot, November 2015) supplemented with the cRAP database (laboratory proteins and dust/contact proteins: http://www.thegpm.org/crap/), and the internal standards defined above (20 230 entries). Peptide mass tolerance was maximum 15 ppm and fragment mass tolerance 0.2 Da. Methylthio (on cysteine) was set as fixed modification, and deamidation (on asparagine and/or glutamine) and oxidation (on methionine) as variable modifications. The enzyme specificity was set to trypsin, with maximum two missed cleavages. Decoy and percolator were enabled. For library size evaluation using the different fractionation methods, expectancy and ion score cut‐off were set to 0.01 and the number of protein families was reported. For SWATH analysis, mzIdentML files were exported with an expectancy cut‐off of 0.01, using the specifications determined in the user guide of the SWATH Acquisition MicroApp 2.0.1 for importing MASCOT results.

SWATH data was processed using the SWATH Acquisition MicroApp 2.0.1 in PeakView 2.2 Software. For each fractionation method, a separate SWATH project was created in which the search results of the respective fractionation library were matched with the unfractionated SWATH data set, the latter being the same for each library. MzIdentML files were imported without specified maximum number of proteins, and shared peptides were excluded. The SWATH Acquisition MicroApp 2.0.1 uses the spectrum that best represents the peptide when peptides are identified multiple times. Retention time alignment was performed with the spiked standard iRT peptides. The processing settings for peak extraction were optimized based on the criterion of number of extracted proteins, peptides and transitions using Lib_DDA (Supporting Information Table 3). The optimal parameters were then applied to the SWATH extractions of all the reference libraries: (i) ten Peptides per Protein, (ii) six Transitions per Peptide, (iii) MASCOT score instead of Peptide Confidence Threshold, (iv) FDR 1%, (v) do not exclude Modified Peptides, (vi) Fix Rank not selected, (vii) five min XIC Extraction Window and (viii) 50 ppm XIC Width. Only unique peptides were used for quantification as shared peptides were not imported. All information (including areas, score, FDR, observed retention time) was exported in .xlsx format for results analysis.

### 2.8 Data analysis

GRAVY scores were determined using http://www.gravy-calculator.de/, and p*I* and molecular weight using http://web.expasy.org/compute_pi/. The SWATH Acquisition Replicates Template was used to evaluate the reproducibility of the transitions based on the biological replicates one to five of diff hESC. Differential protein expression analysis between undiff and diff hESC was performed in MarkerView (Sciex) using a two‐sample t‐test of the normalized protein peak areas based on total area sums. Default MarkerView parameters were used, including 0.0 for missing values and arithmetic sums of transitions/peptides for peptide/protein measurements. Venn diagrams were constructed using http://bioinfogp.cnb.csic.es/tools/venny/index.html. Pathway overrepresentation enrichment analysis was performed using Webgestalt (http://www.webgestalt.org/option.php). For each library, its set of differentially expressed proteins (*p*‐value < 0.05) with a 1.5 fold in‐ or decrease was uploaded and searched against the Wikipathways database. Enrichment analysis was performed after comparison with the list of quantified proteins with the respective library. Benjamini & Hochberg correction was used for multiple testing adjustment, and the significance level was set to 0.05.

## 3 Results and discussion

**Figure 1:**
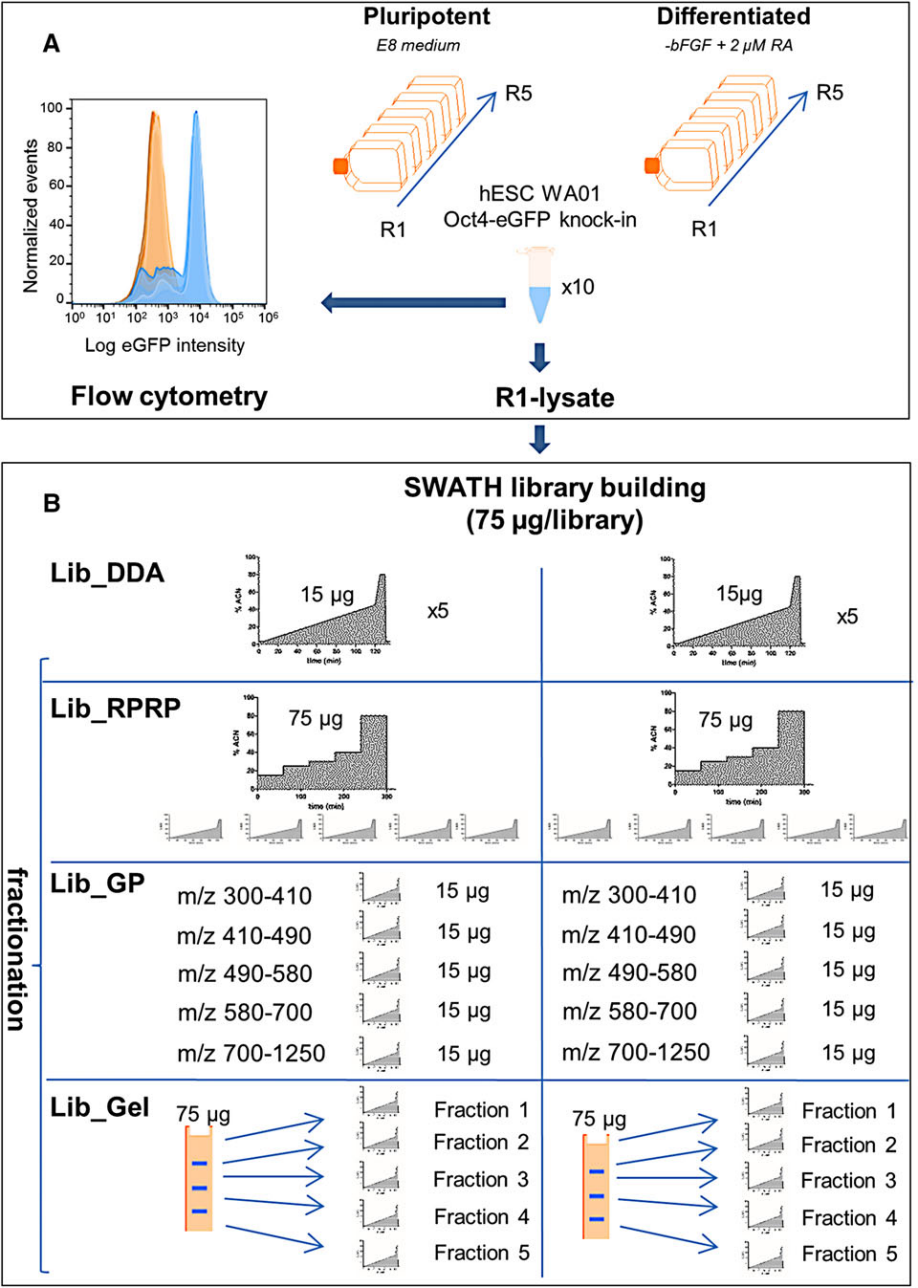
Scheme depicting the experimental workflow. (A) Five biological replicates of undiff and five biological replicates of diff Oct4‐eGFP knock‐in hESC were harvested and their water‐soluble proteome extracted. Representative flow plots of the eGFP detection of cells from the respective populations are shown in blue (undiff) and orange (diff). (B) The different (fractionation) techniques that are compared for SWATH library building: Lib_DDA, Lib_GP, Lib_RPRP and Lib_Gel.

In the most widely employed SWATH workflow, reference libraries are used for the targeted data processing of SWATH samples. Because the coverage and quality of these libraries directly correlate with the interpretation of the results, their creation is considered a primary investment for SWATH users 1, 3, 7, 11. A recent report by Wu et al. confirmed the high peptide identification success of local libraries 1. However, to our knowledge, a comparison of local libraries of different size obtained by different fractionation methods has not been made to date. In this study, local libraries were created using a selection of fractionation methods, and compared to a standard library of replicate DDA injection. At the protein level fractionation was done by means of SDS‐PAGE, at the peptide level by means of high‐pH RP‐HPLC and at the MS‐acquisition level by means of GP fractionation. These fractionation approaches were applied on Oct4‐eGFP knock‐in hESC which were prompted to differentiation using retinoic acid (RA). Loss of pluripotency was monitored by means of eGFP detection using flow cytometry. To minimize extraction bias, the hydrophilic proteome was extracted from five biological replicates of undifferentiated (undiff) hESC and from five biological replicates of (diff) hESC derivates (Fig. 1A). For library building, the five biological replicates were pooled per condition. Lib_DDA consisted of five replicate injections of the pooled sample. For the fractionation libraries, five fractions were created according to the different fractionation methods, followed by DDA acquisition of each fraction (Fig. 1B). Consistent loading amounts, the same number of runs, the same MS‐acquisition method and the same data processing workflow were applied. For SWATH acquisition, the biological replicates were nor pooled, nor fractionated.

### 3.1 Library size and characteristics

**Figure 2:**
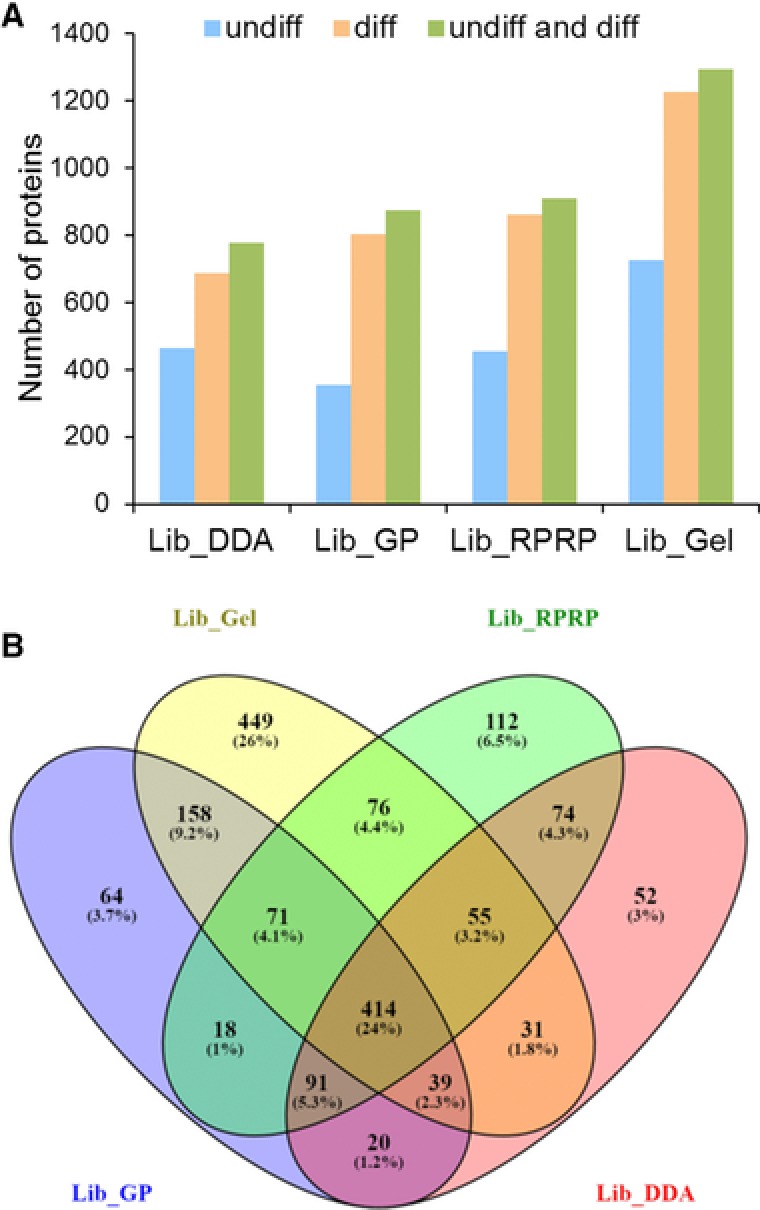
Overview of the different libraries. (A) Bar chart depicting the proteins per library for undiff hESC, diff hESC and a merge of undiff and diff hESC. (B) Venn diagram showing the overlap between identified proteins in Lib_DDA, Lib_GP, Lib_RPRP and Lib_Gel.

First, the different library building strategies were compared for the number of identified proteins (Fig. 2A). After combining identifications from both undiff and diff samples, replicate DDA injection (Lib_DDA) resulted in the identification of 776 proteins. This number increased for GP fractionation (Lib_GP) to 875 proteins, for high‐pH RP‐HPLC fractionation (Lib_RPRP) to 911 proteins and for SDS‐PAGE fractionation (Lib_Gel) to 1293 proteins. Combined, a total of 1688 proteins was identified. The Venn diagram in Fig. 2B shows the overlap among the different libraries. Only 414 proteins were identified with all four fractionation methods, while 52, 64, 112, and 449 proteins were identified exclusively in Lib_DDA, Lib_GP, Lib_RPRP and Lib_Gel, respectively. For the accompanying Venn diagram depicting the number of peptides identified, see Supporting Information Fig. 1. Figure 2A also shows a clear protein shift during differentiation, which confirms the need to include all conditions of a time‐lapse experiment in the reference library that are to be quantified by SWATH. The overlap between undiff and diff hESC for each reference library is included in Supporting Information Fig. 2.

Because each library covered a specific set of proteins (Fig. 2B and Supporting Information Table 4), we assessed whether these differences were related to the hydropathy scale of the proteins (GRAVY score), to their molecular weight or their p*I*. Supporting Information Fig. 3 shows that all libraries displayed a similar distribution of molecular weight and p*I*, and that Lib_Gel displayed a small shift towards more hydrophobic proteins.

### 3.2 SWATH quantification performance

The reference library size can be increased by fractionation, but SWATH runs are generally performed on the unfractionated sample. In a next step, we evaluate the SWATH performance when larger libraries derived from different fractionation methods are used.

#### 3.2.1 Number of peptides and proteins extracted

**Figure 3:**
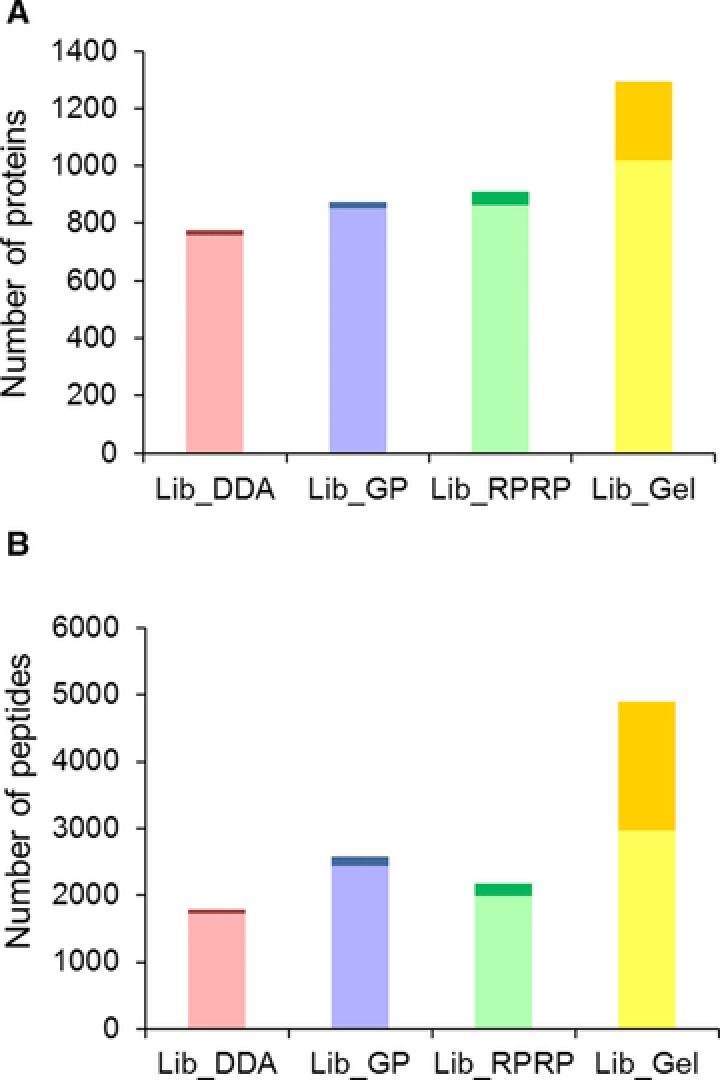
Comparison of library size and extracted quantitative data. (A) Number of proteins in the library (full bar: light + dark) and their extraction (light). (B) Number of peptides targeted (full bar: light + dark) and their extraction at FDR < 1% (light).

The total number of peptides extracted (< 1% FDR) and corresponding proteins from all SWATH samples using the four different reference libraries was examined (Fig. 3A and 3B). Compared to Lib_DDA the fractionation libraries increased the total number of proteins extracted: Lib_DDA < Lib_GP ≈ Lib_RPRP < Lib_Gel. The fractionation libraries also increased the total number of extracted peptides, however the order was different than for the protein level: Lib_DDA < Lib_RPRP < Lib_GP < Lib_Gel. This highlights the high number of peptides extracted using Lib_GP, which could be explained by the fact that GP fractionation focuses on only one fifth of the *m/z* range for the entire MS time, while all other features of the analysis are identical to the SWATH acquisition. Supporting Information Fig. 4 shows the portion of the proteins uniquely identified within each library (Fig. 2B) that were also quantified with that library.

Figure 3 also shows that the increase in library size (targeted peptides and proteins) is not proportional to the increase in the number of extracted peptides and proteins from the SWATH files for all libraries. While Lib_GP and Lib_RPRP efficiently extract the peptides and proteins identified, this does not hold true for Lib_Gel. Although the absolute increase in the number of quantified peptides and proteins is largest for Lib_Gel, this increase only represents half of the increase of proteins and targeted peptides in the library.

Taken together, fractionation proteomics can increase the number of proteins and targeted peptides in the library as well as the amount of quantitative data (number of proteins, peptides, transitions) extracted from the SWATH files, but not all fractionation methods do so proportionally.

#### 3.2.2 Quality of peptide extraction

**Figure 4:**
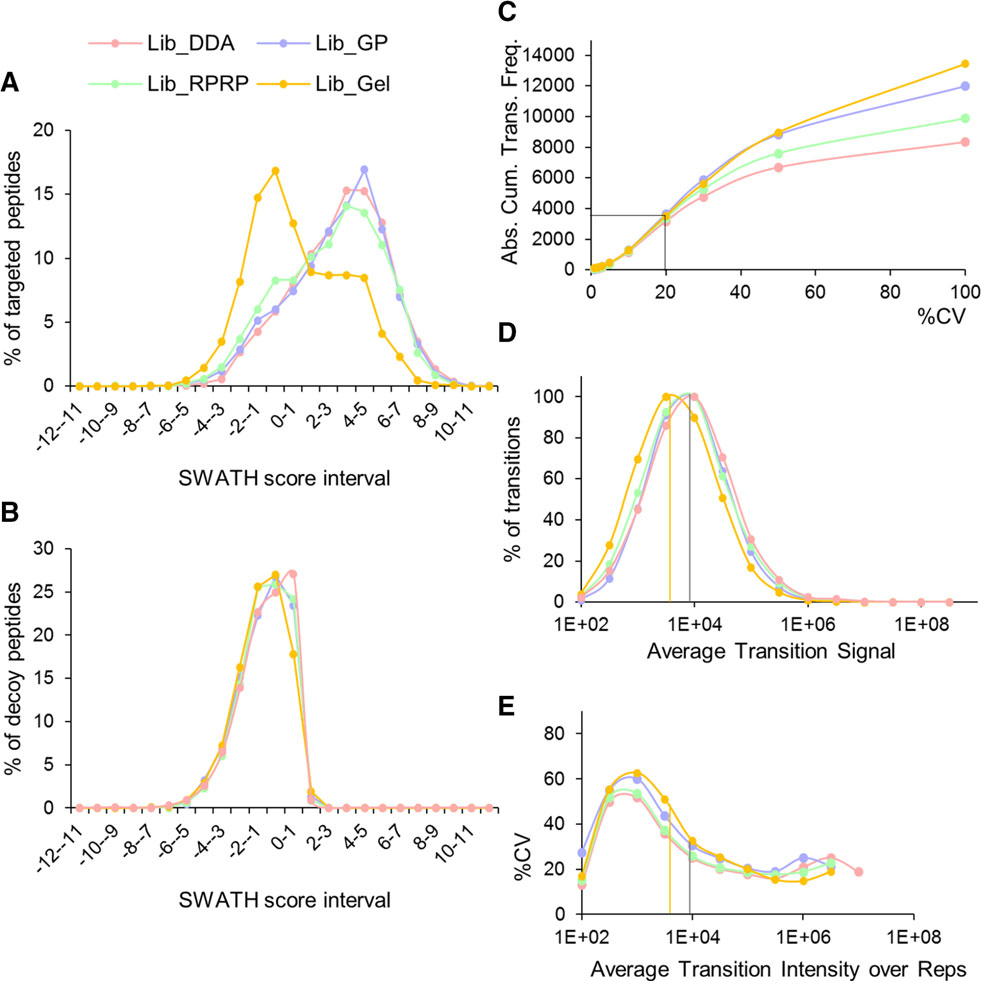
Quality of peptide extraction (A–B) and reproducibility of transitions (C–E). (A) Distribution of the SWATH scores of all targeted peptides (1794 for Lib_DDA, 2579 for Lib_GP, 2172 for Lib_RPRP and 4903 for Lib_Gel) and (B) their decoys in the same SWATH file for the four different libraries. Overlays of the SWATH scores of the targeted and decoy peptides per library can be consulted in Supporting Information Fig. 5. (C–E) The same set of biological SWATH replicates (not repeated injections) from diff hESC (one to five) were matched with the different libraries. (C) Absolute cumulative CV distribution of the transitions. Note that CV 100% includes transitions with CV > 100%. (D) Dynamic range of the transitions extracted. (E) Median CV of the transitions in function of the dynamic range. Vertical bars depict the apex of the transition intensities for the Lib_Gel (yellow) and the other libraries (grey); these lines are projected onto (E) to show that this range intrinsically has higher %CV.

To investigate the reason for this disproportional gain, we evaluated the SWATH scores of the targeted peptides extracted for each reference library (Supporting Information Table 5) 11. Figure 4A shows the distribution of the SWATH scores of the peptides targeted by the four different libraries in the same SWATH file (diff replicate one). This distribution is similar for Lib_DDA, Lib_GP and Lib_RPRP, whereas the apex of the distribution of Lib_Gel is more situated in the low‐scoring part of the distribution. As expected, the distribution of the scores of the decoy peptides was comparable for each library (Fig. 4B). Thus, the majority of the gain in targeted peptides for Lib_Gel as compared to Lib_DDA have scores for peak extraction in the same range as the decoy hits and do not pass the FDR 1% threshold setting defined in the processing settings. One explanation could be that due to increased solubilization and denaturation in the SDS‐PAGE sample preparation protocol (SDS, heat) 21, 22 peptides are created that are not present in the in‐solution digested SWATH samples.

#### 3.2.3 Reproducibility of transitions

Next, we assessed the extraction reproducibility of the transitions from the peptides extracted < 1% FDR 11. For this, the same SWATH runs (biological replicates one to five of diff hESC) were matched against the different libraries. Figure 4C shows the absolute cumulative CV distribution of the transitions extracted by the four libraries. For Lib_DDA, Lib_GP, Lib_RPRP and Lib_Gel 3175, 3637, 3403, and 3533 of the transitions had a CV ≤20%, respectively. According to Fig. 4D and 4E the increased %CV of the supplementary transitions is probably related to the fact that more low‐abundant transitions (10^2^–10^4^) are being quantified (Fig. 4D) which tend to have higher CV in general. This is especially true for Lib_Gel for which the apex of the curve is situated below 10^4^. Figure 4E illustrates how transitions in the range [10^4^–10^6^] give the best CV, irrespective of the library used. Note that these are biological replicates and not repeated injections, partially increasing the overall %CV of the transitions. Finally, we verified whether RT correction of the different DDA runs prior to library building could further improve the results 11, but we found that all runs aligned within ±2.2 min, justifying the choice of a 5 min XIC window for the SWATH data (Supporting Information Table 11). The reduced need for RT corrections during library building compared to Zi et al. is partially due to the shorter gradients used here (90 min) and use of a robust microLC system.

In summary, while fractionation proteomics offers a gain in the extracted transitions, these transitions are not necessarily reproducible (for peptide and protein cumulative plots, see Supporting Information Fig. 6). Thus, although the precision with which low abundant proteins are quantified can be considerably compromised, including an adequate number of biological replicates does allow to obtain a better estimate of the average.

### 3.3 Differential protein expression analysis

Next, we quantified the expression changes in the hESC proteome induced by RA for all reference libraries, and investigated the consistency of detecting differentially abundant proteins. When a t‐test was performed for each protein between undiff and diff samples, this resulted in 363, 420, 358, and 387 proteins that were significantly different (*p*‐value <0.05) with at least 1.5‐fold increase or decrease for Lib_DDA, Lib_GP, Lib_RPRP and Lib_Gel, respectively. These differentially expressed proteins differ substantially (10% common to all libraries, see Supporting Information Fig. 7) which is not surprising based on the limited overlap observed between the different libraries in Fig. 2. Note that the library also defines the background (steady state proteins) against which statistical significance is defined. However, because standard workflows use only one, if any, fractionation technique, one is normally not aware of these differences. Of note, despite these differences, 60–75% of the overrepresented pathways were still equal (data not shown).

**Figure 5:**
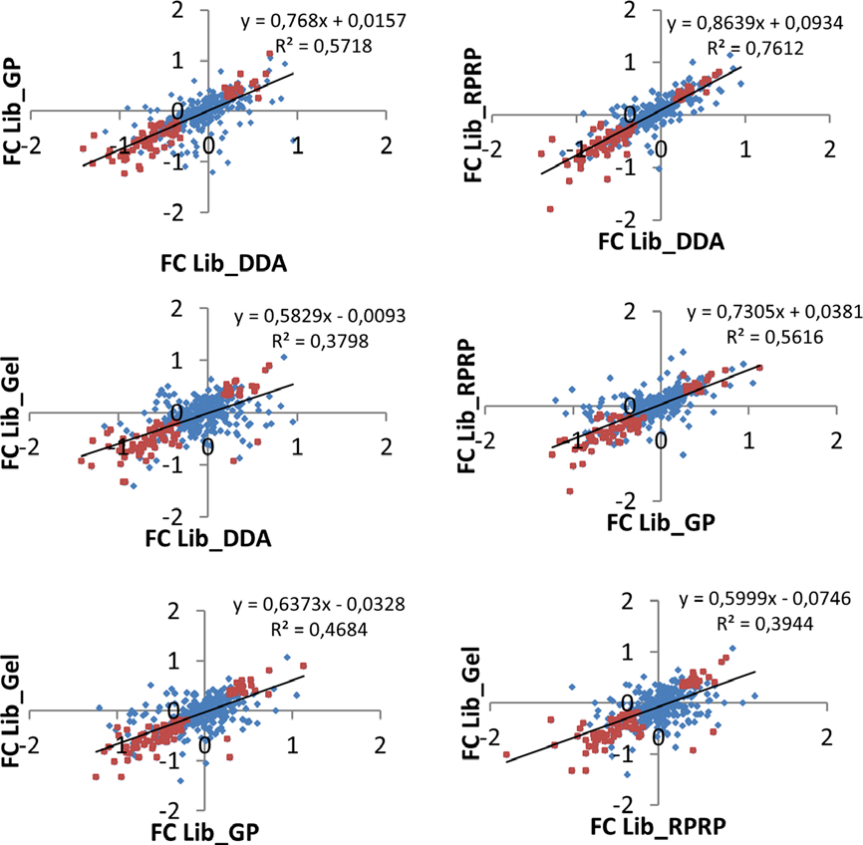
Correlation plots of undiff/diff protein ratios between SWATH quantitative results from each pair of libraries. Blue Diamonds: all commonly quantified proteins; Orange diamonds: commonly quantified differential proteins (*p*‐value < 0.05) with 1.5‐fold in‐ or decrease.

Next, we focused on the pool of commonly identified proteins. Out of these 414 proteins, 385 proteins were also commonly quantified from the same SWATH dataset. When filtering on significant differentially expressed proteins (*p*‐value < 0.05, 1.5 fold change), 203, 215, 193, and 177 proteins were isolated for Lib_DDA, Lib_GP, Lib_RPRP and Lib_Gel, respectively. Of all these proteins, only 83 were deemed significantly up‐ or downregulated in all four libraries. Moreover, two of these common 83 proteins were not consistent in being regulated up or down in all four libraries, even though they were always found to be significant (Supporting Information Table 6). Figure 5 and Supporting Information Fig. 8 show correlation plots of undiff/diff protein ratios between each pair of libraries, highlighting proteins that are differentially quantified (*p*‐value < 0.05, 1.5‐fold change) in common. We hypothesize that through normalization the ratios can considerably be changed depending on the composition of the library. Thus, while correlations between the libraries can be expected, it is clear that the choice of library also has an impact on the selection of statistically significant differential proteins. Especially when focusing on single proteins, for instance biomarkers, as opposed to pathways, this can result in different outcomes. Importantly, because only one library is generally chosen in an experimental setup, future differential proteome analyses on the same biology should not simply use a concordance model (where each protein is assumed to be differential in either study or in none of the studies) but instead use rank order or latent probability vectors (correlation motifs) to compare their list of differently expressed proteins with another one 23. In that case, the importance of the choice of library could be attenuated.

To evaluate the biological relevance of the results, several proteins with particular relevance to hESC biology and ectoderm differentiation were targeted and observed to be significantly different expressed in our experiment (*p* < 0.05, fold change 1.5 up/down, Supporting Information Tables 7–10) 24, 25, 26, 27. Twenty genes are indicated to be associated with undifferentiated hESC by the International Stem Cell Initiative at the mRNA level, six of which constitute a core set of markers to define undifferentiated hESC 28. From this list, DNA (cytosine‐5)‐methyltransferase 3B (*DNMT3B*), protein lin‐28 homolog A (*LIN28*) and podocalyxin (*PODXL*) were observed to be down regulated at the protein level here, which is in agreement with a loss of pluripotency at the mRNA level. The down regulation of green fluorescent protein (GFP), which correlates with the expression of core pluripotency marker Oct4 (*POU5F1*), serves as a validation of the experiment as it was also measured using flow cytometry. Supporting Information Table 11 shows the identification, quantification and statistical analysis (*p*‐value and fold change) of these four proteins with each library, demonstrating that only Lib_GP and Lib_Gel were able detect the differential expression of eGFP.

In summary, Table 1 provides the reader with an overview of the benefits and drawbacks of each fractionation method used for reference library building for SWATH analysis in this study. This table indicates no benefit of replicate DDA injection or high‐pH RP‐HPLC fractionation over GP fractionation or SDS‐PAGE fractionation.

**Table 1:** Overview of the benefits and drawbacks of each fractionation method for local reference library building evaluated in this study

	DDA replicate injection	MS‐acquisition GP fractionation	Peptide RPRP fractionation	Protein SDS‐PAGE fractionation
Cost‐effectiveness	++	++	‐	+
Time investment	++	++	‐	‐
Number of proteins in library	‐	+	+	++
Number of peptides in library (targeted)	‐	++	+	++
Number of extracted proteins	‐	+	+	++
Number of extracted peptides FDR < 1%	‐	++	+	++
Number of extracted proteins CV < 20%	+	+	+	+
Number of extracted peptides CV < 20%	+	+	+	+
Number of extracted transitions CV < 20%	+	+	+	+
Extraction of core pluripotency markers	+	++	‐	++

## 4 Concluding remarks

In conclusion, this study shows that fractionation proteomics increases the size of local reference libraries as well as the number of transitions, peptides, and proteins extracted from the SWATH files compared to standard library building using DDA replicate injection. However, we highlight that this increase in quantitative data is not proportional to the increase in the library size when fractionation methods based on different physicochemical properties are used. Moreover, we point out that the quantitative data obtained from fractionation proteomics at least in part concerns the low abundant, high‐CV region which requires more biological replicates for a more accurate estimation of the average abundance. We also show that different reference libraries built using different fractionation methods can lead to other proteins that are found to be differentially expressed, warranting caution in the interpretation of proteomics results.

The authors have declared no conflict of interest.

## Supporting information

Supplementary Table 1. Determination of m/z ranges for GP fractionation based on replicate DDA injection runs.

Supplementary Table 2. Variable windows calculated using the SWATH Variable Window Calculator (Sciex). No manual intervention.

Supplementary Material

Supplementary Table 4. Proteins identified per reference library.

Supplementary Table 5. SWATH scores of the targeted peptides for each library in all samples.

Supplementary Material

Supplementary Material

Supplementary Material

Supplementary Material
