# Supplementary material for: Comparison of fractionation proteomics for local SWATH library building
Source: Proteomics. 2017 Aug 22;17(15-16):1700052. doi: 10.1002/pmic.201700052 (PMC5601298; doi:10.1002/pmic.201700052)
Supplement: Supplementary file 9 — Supplementary Material [file PMIC-17-na-s009.docx]

Supporting Information

Comparison of fractionation proteomics for local SWATH library building

Elisabeth Govaert^‡^, Katleen Van Steendam^‡^, Sander Willems, Liesbeth Vossaert^¥^, Maarten Dhaenens^¥^, Dieter Deforce^¥^*

Laboratory of Pharmaceutical Biotechnology, Ghent University, Ottergemsesteenweg 460, 9000 Ghent, Belgium

^‡^These authors contributed equally

^¥^These authors contributed equally

**^*^Correspondence**: Prof. Dr. Dieter Deforce, Laboratory of Pharmaceutical Biotechnology, Ghent University, Ottergemsesteenweg 460, 9000 Ghent, Belgium
**Email**: [Dieter.Deforce@UGent.be](mailto:Dieter.Deforce@UGent.be), **Fax**: +32 (0)9 264 81 84

**Contents**

Page 3-4 Details for SDS-PAGE fractionation

Page 5 Details for high-pH RP-HPLC fractionation

Page 6-7 Details for GP fractionation

Page 8 Supplementary Figure 1

Page 9 Supplementary Figure 2

Page 10 Supplementary Figure 3

Page 11 Supplementary Figure 4

Page 12 Supplementary Figure 5

Page 13 Supplementary Figure 6

Page 14 Supplementary Figure 7

Page 15 Supplementary Table 11

**Details for SDS-PAGE fractionation**

- **Gel image**

**
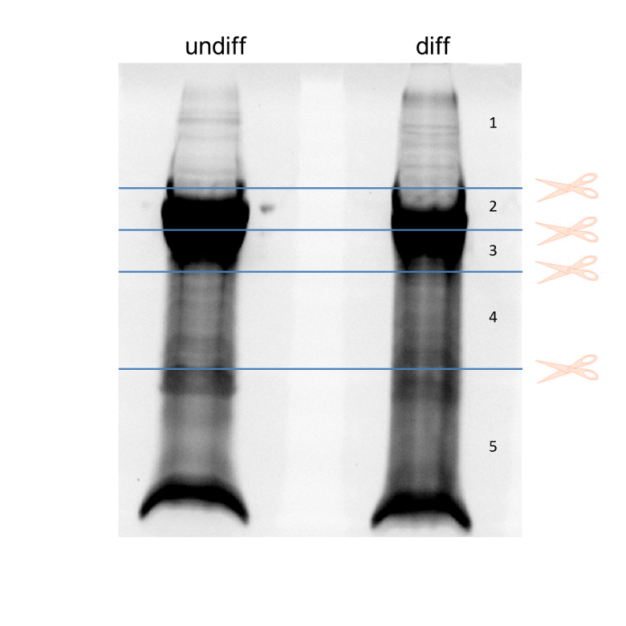
**

- **MS1 profiles of fractions 1-5 for undiff and diff hESC**

Undiff hESC

Diff hESC

**Details for high-pH RP-HPLC fractionation**

- **MS1 profiles of fractions 1-5 for undiff and diff hESC**

Undiff hESC

Diff hESC

**Details for GP fractionation**

- **Determination of the *m/z* range of the five fractions for GP fractionation**

The *m/z* ranges for GP fractionation were determined using the direct support for GP optimization in Progenesis QIP. This optimization was based on the ten DDA runs as pilot samples (five DDA runs of undiff hESC and five DDA runs of diff hESC).

More precisely, at the ‘filter’ stage in the software, the option ‘optimise gas phase fractionation’ can be selected. After specifying the desired number of fractions (here: five), the optimal *m/z* ranges containing approximately the same number of peptides in each fraction are calculated:


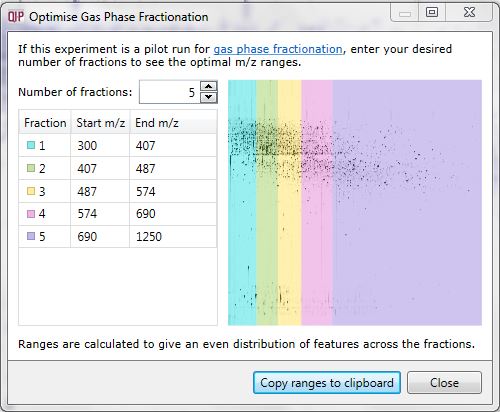


The *m/z* ranges for each DDA run can be found in Supplementary Table 1. As they were for all DDA runs (undiff and diff) very much alike, a consensus was chosen for the GP fractionation of both undiff and diff hESC, which can also be found in Supplementary Table 1.

- **MS1 profiles of fractions 1-5 for undiff and diff hESC**

Undiff hESC

Diff hESC

**Supplementary Figure 1.** Venn diagram showing the overlap between identified peptides (expectancy cut-off 0.01) in Lib_DDA, Lib_GP, Lib_RPRP and Lib_Gel.

**
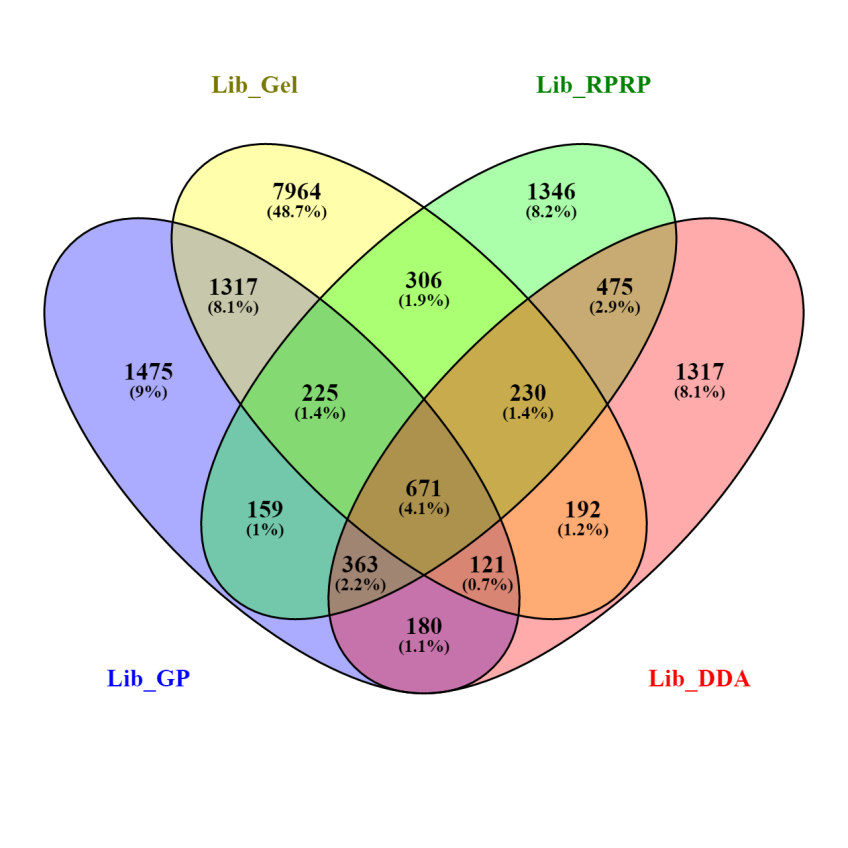
**

**Supplementary Figure 2.** Overview of the overlap (protein families) between undiff and diff hESC for each reference library: Lib_DDA, Lib_GP, Lib_RPRP and Lib_Gel.


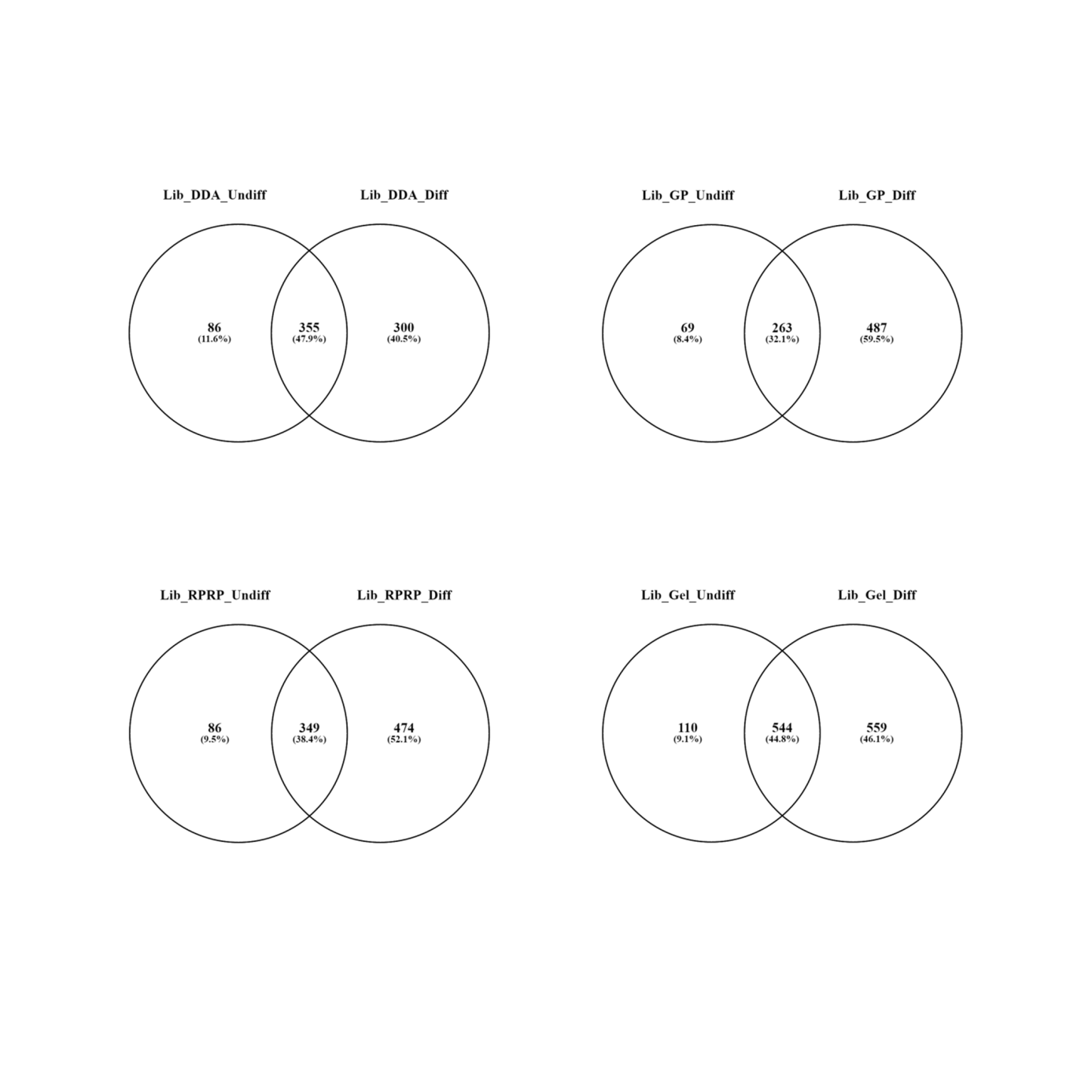


**Supplementary Figure 3.**  Molecular weight, p*I* and GRAVY score of the proteins identified in each library.


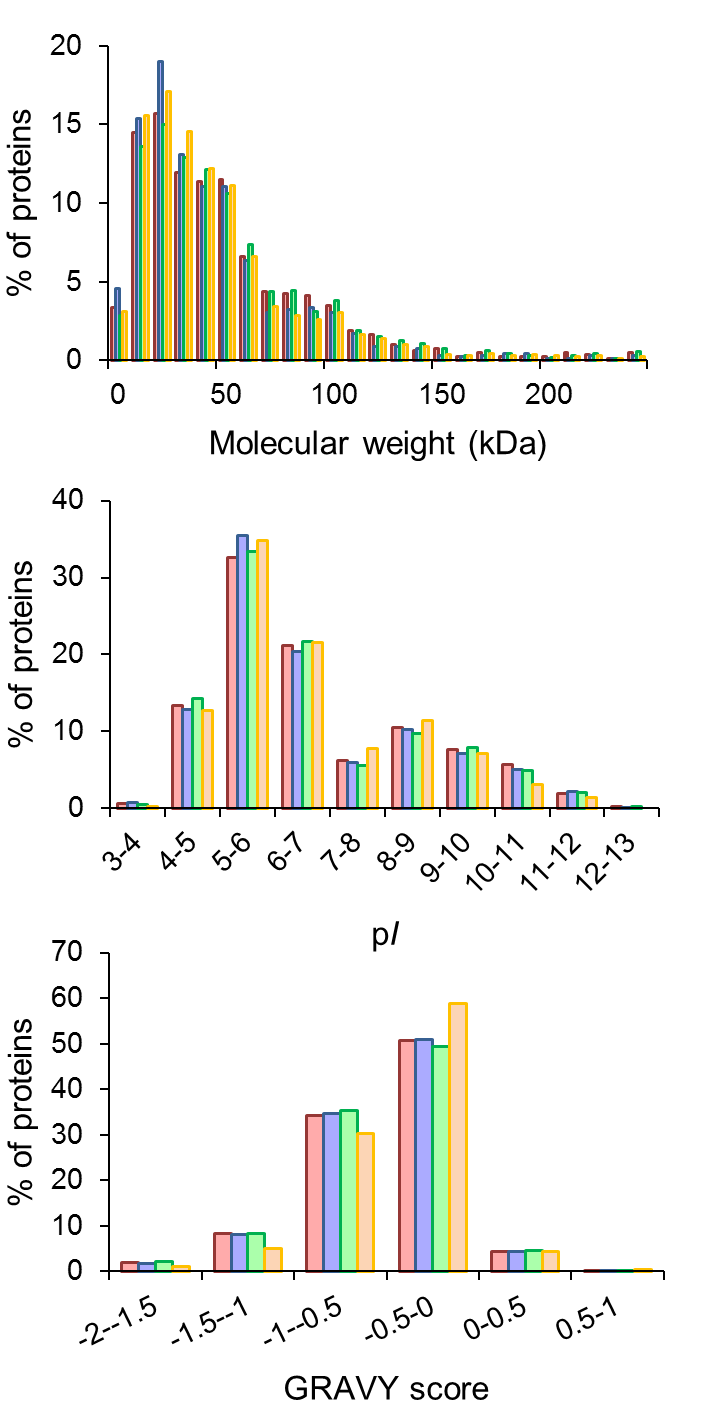


**Supplementary Figure 4.** Percentage of the uniquely identified proteins (full bar: light + dark) for each reference library that is also quantified (light) with that library.


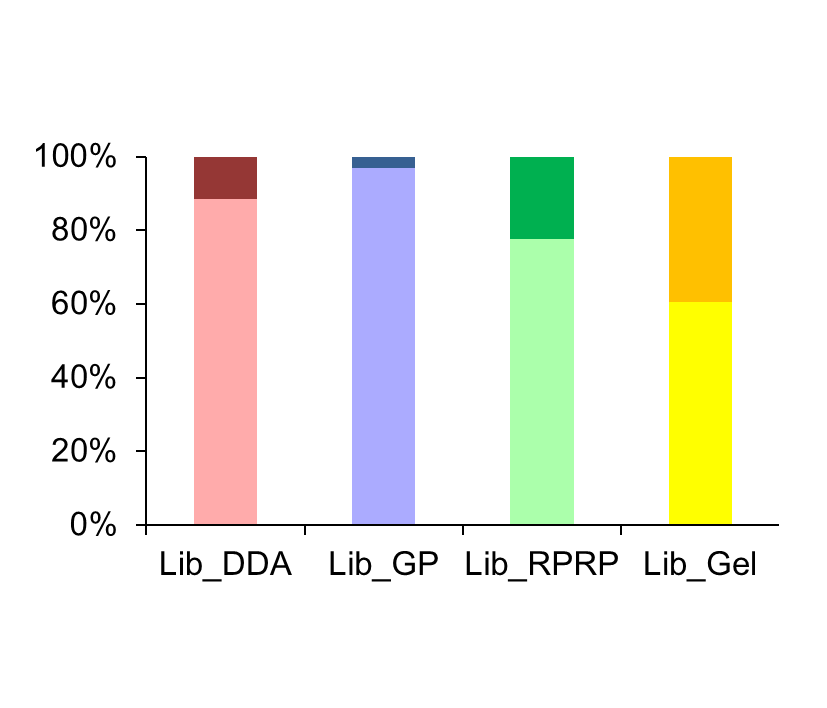


**Supplementary Figure 5.** Overlay of SWATH score distributions of decoy (red) and targeted (blue) peptides extracted from diff hESC replicate one using the different reference libraries: A, Lib_DDA; B, Lib_GP; C, Lib_RPRP; D, Lib_Gel.


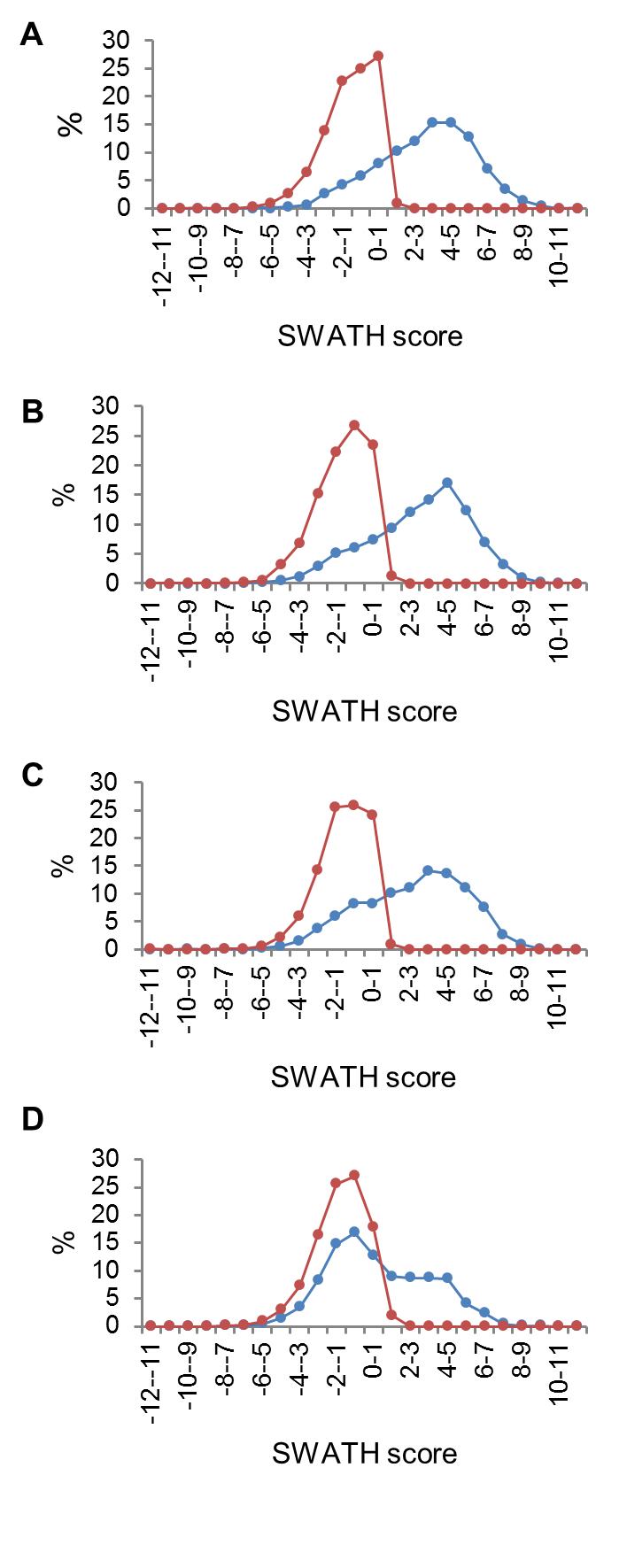


**Supplementary Figure 6.** (A) Absolute cumulative CV distribution of the peptides extracted (< 1% FDR) and (B) the related proteins. Note that CV 100 includes peptides and proteins with CV > 100. Color code: red, Lib_DDA; green, Lib_RPRP; blue, Lib_GP and orange, Lib_Gel.


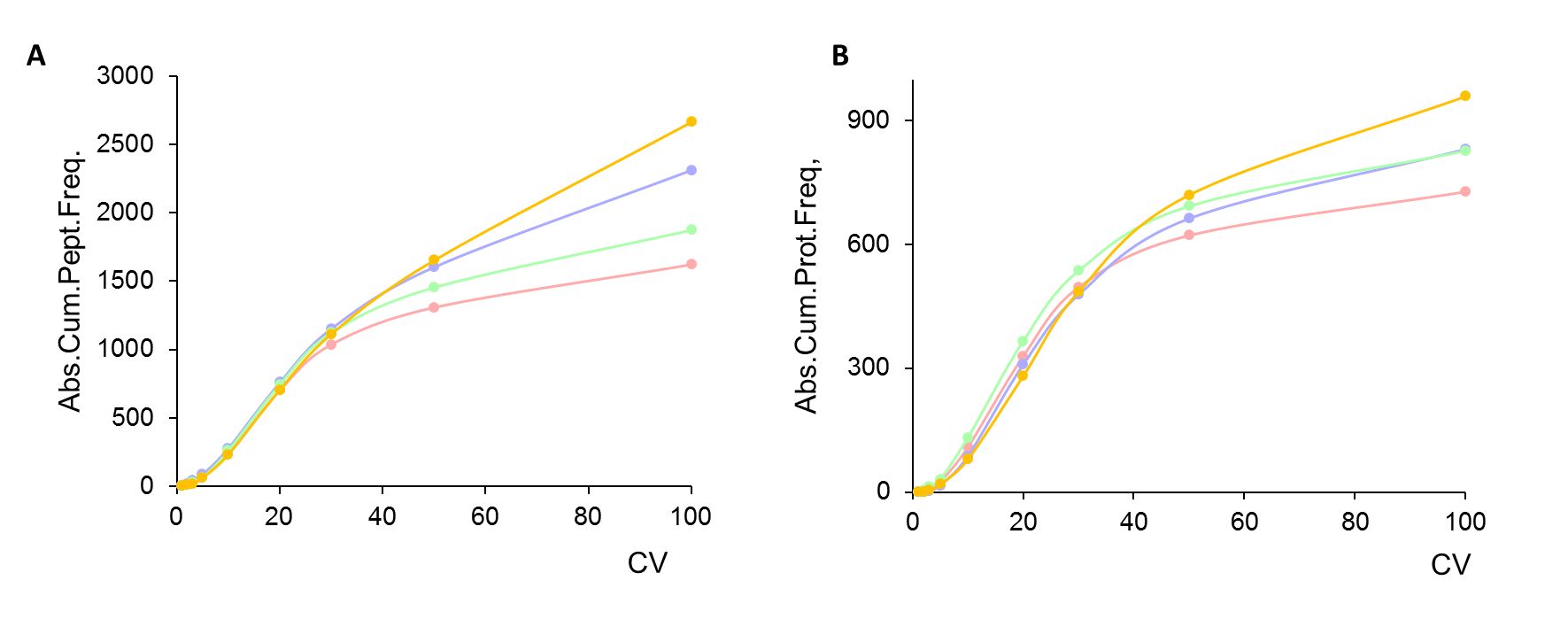


**Supplementary Figure 7.** Overlap of differentially expressed proteins (p < 0.05; 1.5-fold increase or decrease) between the different libraries.


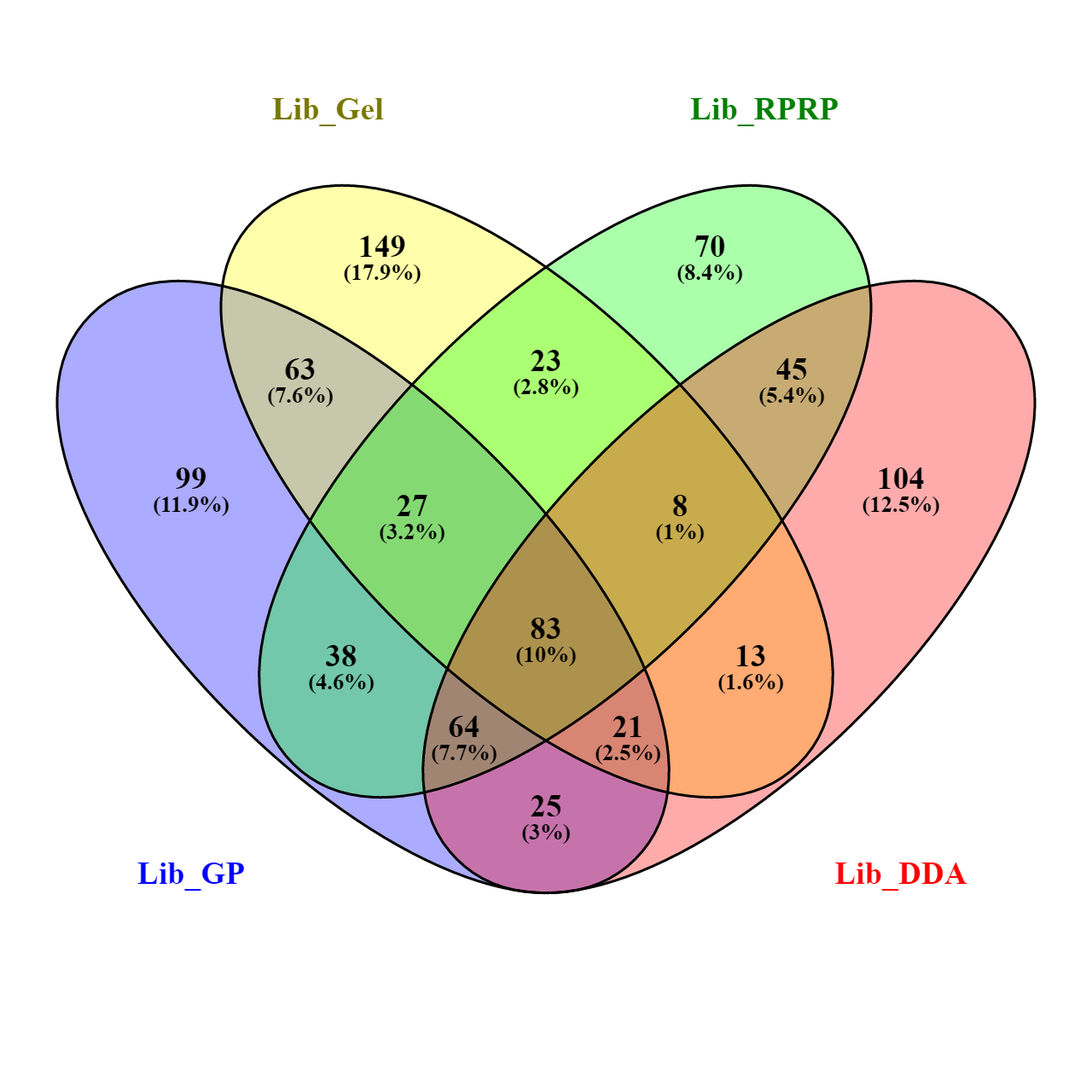


**Supplementary Table 11.** Four genes related to undifferentiated hESC detected at the protein level in this study. L = identified in library, Q = quantified, P = p-value, and F = fold change.

|  | **Lib_DDA** | | | | **Lib_GP** | | | | **Lib_RPRP** | | | | **Lib_Gel** | | | |
| --- | --- | --- | --- | --- | --- | --- | --- | --- | --- | --- | --- | --- | --- | --- | --- | --- |
|  | **L** | **Q** | **P** | **F** | **L** | **Q** | **P** | **F** | **L** | **Q** | **P** | **F** | **L** | **Q** | **P** | **F** |
| ***DNMT3B*** | yes | yes | 2.3E-04 | 21.6 | yes | yes | 5.8E-05 | 48.7 | no | N/A | N/A | N/A | yes | yes | 2.4E-02 | 19.3 |
| ***LIN28*** | yes | yes | 1.1E-01 | 2.1 | yes | yes | 8.6E-02 | 2.7 | yes | yes | 8.5E-02 | 2.6 | yes | yes | 3.6E-02 | 3.1 |
| ***PODXL*** | yes | yes | 1.4E-01 | 7.1 | yes | yes | 1.3E-01 | 8.7 | yes | yes | 4.5E-04 | 7.6 | yes | yes | 2.4E-02 | 11.7 |
| ***GFP*** | no | N/A | N/A | N/A | yes | yes | 4.2E-02 | 2.2 | no | N/A | N/A | N/A | yes | yes | 1.2E-02 | 3.4 |
